# Comprehensive analysis of prognostic alternative splicing signature in cervical cancer

**DOI:** 10.1186/s12935-020-01299-4

**Published:** 2020-06-08

**Authors:** Dong Ouyang, Ping Yang, Jing Cai, Si Sun, Zehua Wang

**Affiliations:** 1 Department of Obstetrics and Gynecology, Union Hospital, Tongji Medical College, Huazhong University of Science and Technology, Wuhan, 430022 China; 2 Department of Obstetrics and Gynecology, Akesu Hospital of Traditional Chinese Medicine, Akesu, China; 3 Department of Obstetrics and Gynecology, First Affiliated Hospital, School of Medicine, Shihezi University, Shihezi, China

**Keywords:** AS, Nomogram, Prognosis, Splicing factor, The Cancer Genome Atlas, Cervical cancer

## Abstract

**Background:**

Alternative splicing (AS) is a key factor in protein-coding gene diversity, and is associated with the development and progression of malignant tumours. However, the role of AS in cervical cancer is unclear.

**Methods:**

The AS data for cervical squamous cell carcinoma and endocervical adenocarcinoma (CESC) were downloaded from The Cancer Genome Atlas (TCGA) SpliceSeq website. Few prognostic AS events were identified through univariate Cox analysis. We further identified the prognostic prediction models of the seven subtypes of AS events and assessed their predictive power. We constructed a clinical prediction model through global analysis of prognostic AS events and established a nomogram using the risk score calculated from the prognostic model and relevant clinical information. Unsupervised cluster analysis was used to explore the relationship between prognostic AS events in the model and clinical features.

**Results:**

A total of 2860 prognostic AS events in cervical cancer were identified. The best predictive effect was shown by a single alternate acceptor subtype with an area under the curve of 0.96. Our clinical prognostic model included a nine-AS event signature, and the c-index of the predicted nomogram model was 0.764. SNRPA and CCDC12 were hub genes for prognosis-associated splicing factors. Unsupervised cluster analysis through the nine prognostic AS events revealed three clusters with different survival patterns.

**Conclusions:**

AS events affect the prognosis and biological progression of cervical cancer. The identified prognostic AS events and splicing regulatory networks can increase our understanding of the underlying mechanisms of cervical cancer, providing new therapeutic strategies.

## Background

Cervical cancer is one of the most common malignancies of the female genital tract. It is the fourth leading cause of cancer-related death in females, with an estimated 311,365 deaths worldwide in 2018 [1]. The risk of death in females with cervical cancer is higher in low-income countries (0.9%) than in high-income countries (0.3%) [2]. Despite a series of advances in the prevention, screening, and treatment of cervical cancer (e.g., modern radiotherapy techniques and targeted therapy), the effectiveness of treatment against cervical cancer has not significantly improved [3, 4]. In China, the overall morbidity and mortality associated with cervical cancer has steadily increased from 1991 to 2013, and it is predicted to continue to rise in the future [5]. Following metastasis or recurrence of cervical cancer, the disease is linked to a poor prognosis, with a 5-year overall survival (OS) rate of only 17% [6]. Hence, identifying novel therapeutic targets and survival-associated biomarkers is essential to enhance the therapeutic effect in cervical cancer.

Recent progress made in the field of large-scale multi-omics research provides a new perspective for the study of the occurrence and development of cancer through systems biology. Cervical cancer is considered a virus-driven cancer, and the human papilloma virus has been widely recognized as a causative factor for cervical cancer. Early infection with the human papilloma virus may be merely an induced event. Genome alterations (e.g., gene fusion, non-coding RNAs, copy number variation, DNA methylation, and somatic DNA mutations) will eventually lead to the malignant transformation of cervical epithelial cells [7–11]. Previous prospective studies mainly focused on alterations at the transcriptome and epigenetic levels. However, a systematic analysis of post-transcriptional splicing isoforms (alternative splicing [AS]) in cervical cancer is currently lacking.

AS is a peculiar biological process in eukaryotes, by which a single gene can generate different protein isomers to drive proteome diversity. More than 94% of genes are alternatively spliced in humans; moreover, the prevalence and hallmarks of alternative splicing are considerably different [12–14]. Cancer cells use this mechanism to produce aberrant proteins with abnormal functional domains that lead to tumorigenesis [15–17]. These alterations in domains can result in complex remodelling and protein–protein interactions in cancers. Some relevant oncogenic splicing variants can directly regulate processes related to cancer stem cell biology and epithelial-to-mesenchymal transition in tumours [18]. It has been shown that there are 30% more AS events in tumours as compared to healthy samples [19]. Therefore, cancer-specific AS may act as a diagnostic and prognostic biomarker, as well as ultimately guide treatment.

The Cancer Genome Atlas (TCGA) project collects RNA sequencing (RNA-Seq) data from several different types of cancer, providing a rich resource for studying aberrant RNA splicing in cancer. Some investigations have used TCGA RNA-Seq data to systematically study cancer-related AS events, including ovarian cancer [20], kidney renal clear-cell carcinoma [21], bladder cancer [22], prostate cancer [23], colorectal cancer [24, 25], and lung cancer [26]. In our study, a global analysis of survival-associated AS events in cervical squamous cell carcinoma and endocervical adenocarcinoma (CESC) was conducted. Using the different splicing patterns of nine genes, we constructed a predictive model that can stratify risk for patients with CESC. A regulatory network was established that included prognostic splicing factors and AS events. In addition, we established a predictive nomogram that combined our nine-AS event signature with clinical pathological risk factors in patients with CESC. A comprehensive analysis of prognostic AS events can assist us in better understanding their potential functions in tumour biology, identifying reliable prognostic biomarkers, and developing new treatments for cervical cancer.

## Methods

### Data curation process

**Fig. 1:**
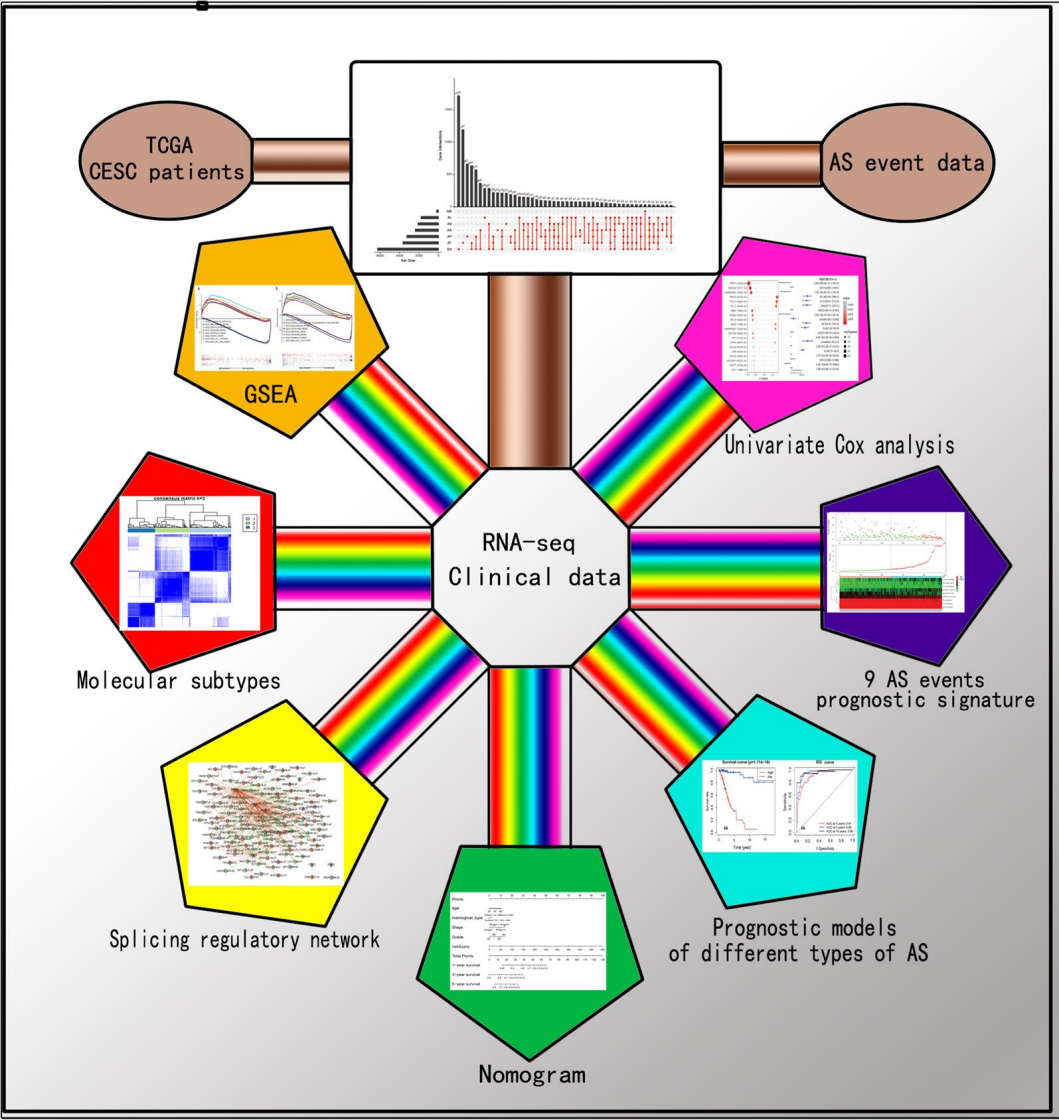
Flowchart for profiling the prognostic alternative splicing signature of CESC. Alternative splicing data were downloaded from the TCGASpliceSeq database. We identified survival-associated AS events in CESC and further identified a signature consisting of nine prognostic AS events. Then, we constructed a splicing regulatory network between prognostic splicing factors and AS events in the CESC cohort. Next, we built a predictive nomogram. Finally, we divided cervical cancer patients into three molecular subtypes based on nine alternative splicing events

We downloaded the level-3 RNA-Seq data and corresponding clinical data for the CESC cohort from the TCGA database (May 2019, https://portal.gdc.cancer.gov/). The AS event data for CESC were obtained from the TCGASpliceSeq database (http://projects.insilico.us.com/TCGASpliceSeq/) [27]. Because these data are available to the public, there was no requirement for approval by an ethics committee. We fully assessed the availability of clinical information. During our research, a few patients were excluded because they met the following criteria: (i) Death occurring within 1 month, or death due to other illnesses and accidents; and (ii) Lack of complete clinical features (e.g., age, grade, pathological stage, and survival data). The percent spliced in (PSI) value can be used to quantify each AS event, which is the ratio of normalized reads indicating the presence of a transcript element versus the total normalized reads for that event, with a rating from 0 to 1. We set the screening criteria (samples with PSI value > 75% and average PSI value ≥ 0.05) to obtain the AS profile for each patient with CESC. A total of 243 patients with complete AS event data and clinical data were included in our analysis. The clinical features of the patients are summarized in Table 1, and a flowchart of this study is shown in Fig. 1.

**Table 1:** The main demographic, clinical, and pathological characteristics of the 243 patients with CESC

Characteristic	Survival (n = 187)	Death (n = 56)	P value*
Age (years)			
≤ 60	163 (87.2)	42 (75.0)	0.047
> 60	24 (12.8)	14 (25.0)	
Race			
White	132 (70.6)	41 (73.2)	0.473
Black or African American	20 (10.7)	7 (12.5)	
Asian	16 (8.6)	1 (1.8)	
Other	6 (3.2)	3 (5.4)	
NA	13 (7.0)	4 (7.1)	
Histological type			
Adenosquamous	4 (2.1)	1 (1.8)	
Cervical squamous cell carcinoma	157 (84.0)	49 (87.5)	
Endocervical adenocarcinoma	26 (13.9)	6 (10.7)	
AJCC stage			
Stage I	113 (60.4)	34 (60.7)	0.002
Stage II	41 (21.9)	6 (10.7)	
Stage III	24 (12.8)	8 (14.3)	
Stage IV	4 (2.1)	8 (14.3)	
NA	5 (2.7)	0 (0.0)	
Grade			
G1	14 (7.5)	1 (1.8)	0.342
G2	78 (41.7)	29 (51.8)	
G3	80 (42.8)	19 (33.9)	
G4	1 (0.5)	0 (0.0)	
GX	14 (7.5)	7 (12.5)	

*CESC* cervical squamous cell carcinoma and endocervical adenocarcinoma, *AJCC* American Joint Committee on Cancer

*P value refers to the level of significance in the χ2 test

### Identification of prognostic AS events and survival analysis

AS events include the following seven types (Additional file 1a): alternate acceptor (AA) site, alternate donor (AD) site, alternate promoter (AP), alternate terminator (AT), retained intron (RI), exon skip (ES), and mutually exclusive exons (ME). Cox univariate analyses assessed AS events of various types that may affect the prognosis of patients with CESC based on OS. The least absolute shrinkage and selection operator (LASSO) regression method was used to perform the dimensionality reduction analysis of the prognostic AS events identified in previous analyses. LASSO sub-selects prognostic AS events for CESC patients by providing a penalty proportional to the contraction of the regression coefficient. In addition, the Cox proportional hazards regression model was adopted to further construct predictive models of prognostic AS events of the seven types. The calculation formula is as follows:

$${\text{Risk}}\;{\text{score}}\;{ = }\;\sum\nolimits_{\text{i}}^{\text{n}} {{\text{PSIi}}\,*\;\upbeta} {\text{i}}$$

With the model classifiers, patients with CESC can be divided into high- and low-risk groups. Kaplan–Meier curves were used to verify whether the predictive models can effectively distinguish patients from these two groups. Receiver operating characteristic (ROC) curves were used to further assess the prediction for the 3-, 5-, and 10-year survival rate using these predictive models via the time ROC package in R (version 3.5.1).

### Construction of the prognostic model

We conducted a global scan analysis of all survival-associated AS events using the LASSO-Cox method to preserve the prognostic value and reduce the molecular features in the model. The R package “glmnet” was used for LASSO-Cox analysis to further study critical AS events. Dimensionality reduction was further performed using the Cox proportional hazards regression model. Finally, a survival-associated AS event signature was constructed and used to calculate the risk score for each patient with cervical cancer. With the AS event signature and the median risk score used as the cut-off value, patients with cervical cancer can be divided into high- and low-risk groups. The Kaplan–Meier and ROC curves were used to evaluate the prognostic and predictive accuracy of the model. All analyses were performed using the R package (survival and survival ROC).

### Establishment of the nomogram

We constructed a clinical application model for predicting the survival rate of patients with CESC. We combined patient clinical information (e.g., tumour stage, grade, and histological types) and patient risk scores using the Cox univariate analysis and Cox multivariate regression model to evaluate independent risk predictors for patients with cervical cancer. In addition, we constructed a nomogram to predict survival using the prognostic risk score prediction model and clinicopathologic risk factors. The “rms” package of R (version 3.5.1) was utilized to visualize the nomogram and calibration plots. The 45° line of calibration plots represents the best predictive effect, and the C-index was used to assess the predicted accuracy of the nomogram.

### Construction of a splicing correlation network

We constructed a regulatory network including splicing factors and prognostic AS events to investigate the effects of currently known splicing factors on these survival-associated AS events. Through a literature search and Internet resources, we listed 404 human splicing factor genes [28]. We obtained the expression of the aforementioned splicing factors from the level 3 RNA-seq data downloaded from the TCGA database. We subsequently analysed the correlation between the splicing factors and these prognostic AS events using Spearman’s rank test. The oncogenic characteristics of HPV are derived from the oncoproteins E6 and E7 that act to inhibit p53 tumour suppressors. TP53 is a protein-coding gene of oncoprotein P53, and its expression also reflects the risk of HPV infection. We compared the correlation between the TP53 gene and the hub splicing factors. Adjusted P-values < 0·05 denote statistical significance. In this manner, we constructed a regulatory network that was visualized using Cytoscape (version 3.6.0) software and included splicing factors and prognostic AS events. The patients in the TCGA cohort were divided into high- and low-expression groups according to the expression level of the hub splicing factor to further understand the potential mechanism of hub splicing factors in the network. Subsequently, a gene set enrichment analysis was performed to examine the Kyoto Encyclopedia of Genes and Genomes pathway associated with the two groups [29].

### Evaluation of the correlation with clinical features

Unsupervised classification of the CESC cohort was conducted through k-means clustering on prognostic AS events of the identified predictive models. We used the R package “ConsensusClusterPlus” to implement an unsupervised consensus approach and obtain a robust classification [30]. Consensus Clustering is an algorithm that can be used to identify clusters and their numbers in data sets, such as microarray gene expression. We combined the consistency matrix heat map, the consistent cumulative distribution function graph, and the Delta Area Plot to determine the number of clusters. Consensus molecular subtyping of CESC was accomplished according to the PSI value of AS events in the prognostic model. Principal component analysis (PCA) is a particularly successful feature extracting a dimensionality-reducing algorithm that can be used to facilitate data exploration and visualization. We performed a PCA of CESC patients with prognostic AS event variables in the model and visualized the molecular subtypes of patients. Finally, we used the Chi square test and logistic regression to assess the correlation between clusters and clinical outcomes.

## Results

### Profiles of AS events in the CESC cohort

A total of 41,776 AS events for 9960 genes were identified in 243 patients with CESC, indicating that the average number of AS events for each gene exceeds four. The frequency of ES occurrence was the highest among the seven AS types. In detail, we observed 3424 AAs of 2398 genes, 8395 ATs of 3664 genes, 8066 APs of 3258 genes, 3017 ADs of 2106 genes, 2723 RIs of 1800 genes, 15,942 ESs of 6277 genes, and 209 MEs of 202 genes (Additional file 1b). Each AS event was assigned a unique code for a more accurate description. For example, in the code for RPP13-22038-AA, RPP13 represents the name of the parent gene, AA indicates the splicing type, and 22038 denotes the ID number in the dataset. The intersections between the AS types were visualized using an UpSet plot (Additional file 1c).

### Prognostic AS events in the CESC cohort

Univariate Cox analysis was used to identify 2860 AS events that were significantly associated with OS (P < 0·05). The top 20 AS events significantly associated with these seven types are shown in Additional file 2a–g. A gene may have multiple prognostic AS events associated with CESC. Thus, the seven types of prognostic AS events in CESC are shown using UpSet plots (Additional file 2 h). Subsequently, the LASSO-Cox method and the Cox proportional hazards regression model were applied to globally analyse prognostic AS events in the seven types, and independently analyse the established prognostic models of the various types. The screened survival-associated AS events of the seven different types from this model were merged to construct the final prognostic model.

**Fig. 2:**
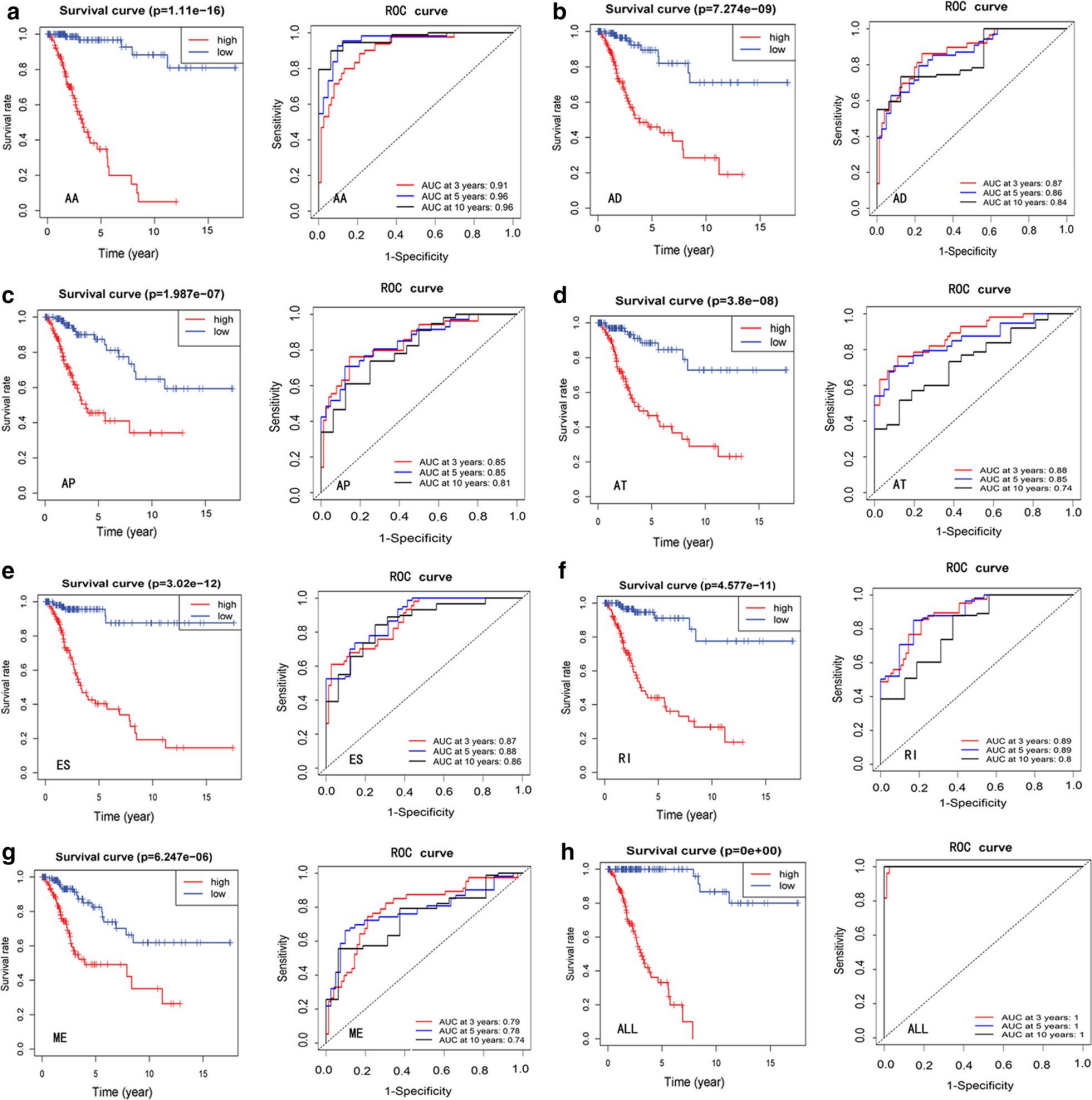
Kaplan–Meier and ROC curves of the seven prognostic models established with different types of AS in the CESC cohort. **a**–**g** Kaplan–Meier and ROC curves describe the survival probabilities over time for predicting prognosis of seven types of AS events with high- and low-risk groups, respectively. **h** Kaplan–Meier and ROC curves describe the combination of all seven AS events for predicting survival probabilities over time

In our study, all the different prognostic models established using the seven types of AS events indicated significant effects in predicting the prognosis of patients with CESC. Among them, the strongest predictive effect was exerted by the prediction model established using the single AA subtype (Fig. 2a–g), with an area under the curve (AUC) of 0·96 (5-year survival). Obviously, the final prognostic model exhibited a predictive effect that was more enhanced than any other single-type splice pattern. The ROC curve was perfect, with an AUC (5-year survival) of 1, followed by the AA and RI models with AUC (5-year survival) of 0.96 and 0.89, respectively (Fig. 2a–h).

### Identification of a nine-AS event prognostic signature in CESC

**Fig. 3:**
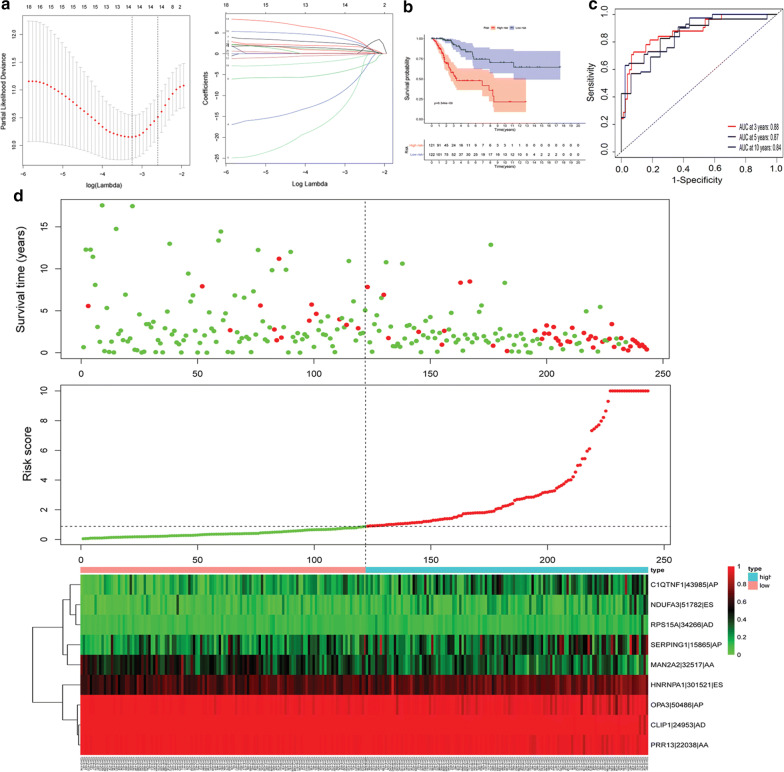
A nine-AS events prognostic signature in CESC. **a** LASSO coefficient profiles of survival-associated AS events and ten-time cross-validation for tuning parameter selection in the LASSO model. **b** Kaplan–Meier analysis for overall survival of CESC patients. **c** ROC curve in the predicted groups (high and low-risk groups) by the nine-AS events signature in the CESC cohort. **d** Risk score distribution of nine-AS events signature in the TCGA cohort including risk scores, survival status and heat map of the nine-AS events PSI profiles

A 14-AS event signature was obtained through the global analysis of all prognostic AS events using the LASSO-Cox method (Fig. 3a). We further reduced the dimensionality of these data. Finally, a nine-AS event signature was identified as a predictor of survival in cervical cancer through the Cox proportional hazards regression model (Table 2). In the multivariate Cox analysis, regression coefficients were weighted for the nine AS events, and a linear prediction model was established. The risk score was calculated as follows: risk score = (1.7164 × PSI of C1QTNF1-43985-AP) + (− 16.0778 × PSI of OPA3-50486-AP) + (− 24.6113 × PSI of CLIP1-24953-AD) + (4.6100 × PSI of HNRNPA1-301521-ES) + (− 9.3599 × PSI of PRR13-22038-AA) + (5.0443 × PSI of NDUFA3-51782-ES) + (2.1513 × PSI of SERPING1-15865-AP) + (8.1402 × PSI of RPS15A-34266-AD) + (− 2.9594 × PSI of MAN2A2-32517-AA). Based on this model, we calculated the risk score for each patient and divided the patients into high- and low-risk groups (Fig. 3b). The low-risk group exhibited a more significant survival benefit (Fig. 3b). In addition, we visualized the relationship between risk scores and cancer-related deaths (Fig. 3d). As the risk score increases, the heat map shows the PSI changes for the nine AS events (Fig. 3d). Our results revealed that 3-year survival of the nine-AS event prediction model yielded an AUC value of 0.88 (Fig. 3c), indicating its high prediction efficiency.

**Table 2:** Nine AS events associated with the OS of patients with CESC

AS events	Coefficient	HR	HR.95L	HR.95H	P value
C1QTNF1-43985-AP	1.7165	5.564908	0.591273	52.37548	0.133464
OPA3-50486-AP	− 16.0778	1.04E-07	2.11E-10	5.13E-05	3.72E-07
CLIP1-24953-AD	− 24.6113	2.05E-11	2.02E-17	2.08E-05	0.000487
HNRNPA1-301521-ES	4.6100	100.4831	0.777403	12,987.93	0.063104
PRR13-22038-AA	− 9.3599	8.61E-05	1.42E-09	5.232286	0.095813
NDUFA3-51782-ES	5.0443	155.1364	9.500676	2533.221	0.0004
SERPING1-15865-AP	2.1513	8.595978	1.75865	42.01565	0.007877
RPS15A-34266-AD	8.1402	3,429.715	9.790151	1,201,508	0.006466
MAN2A2-32517-AA	− 2.9594	0.051851	0.005065	0.530781	0.012642

*AS* alternative splicing, *OS* overall survival, *CESC* cervical squamous cell carcinoma and endocervical adenocarcinoma, *HR* hazard ratios

### Construction and evaluation of the nomogram

**Fig. 4:**
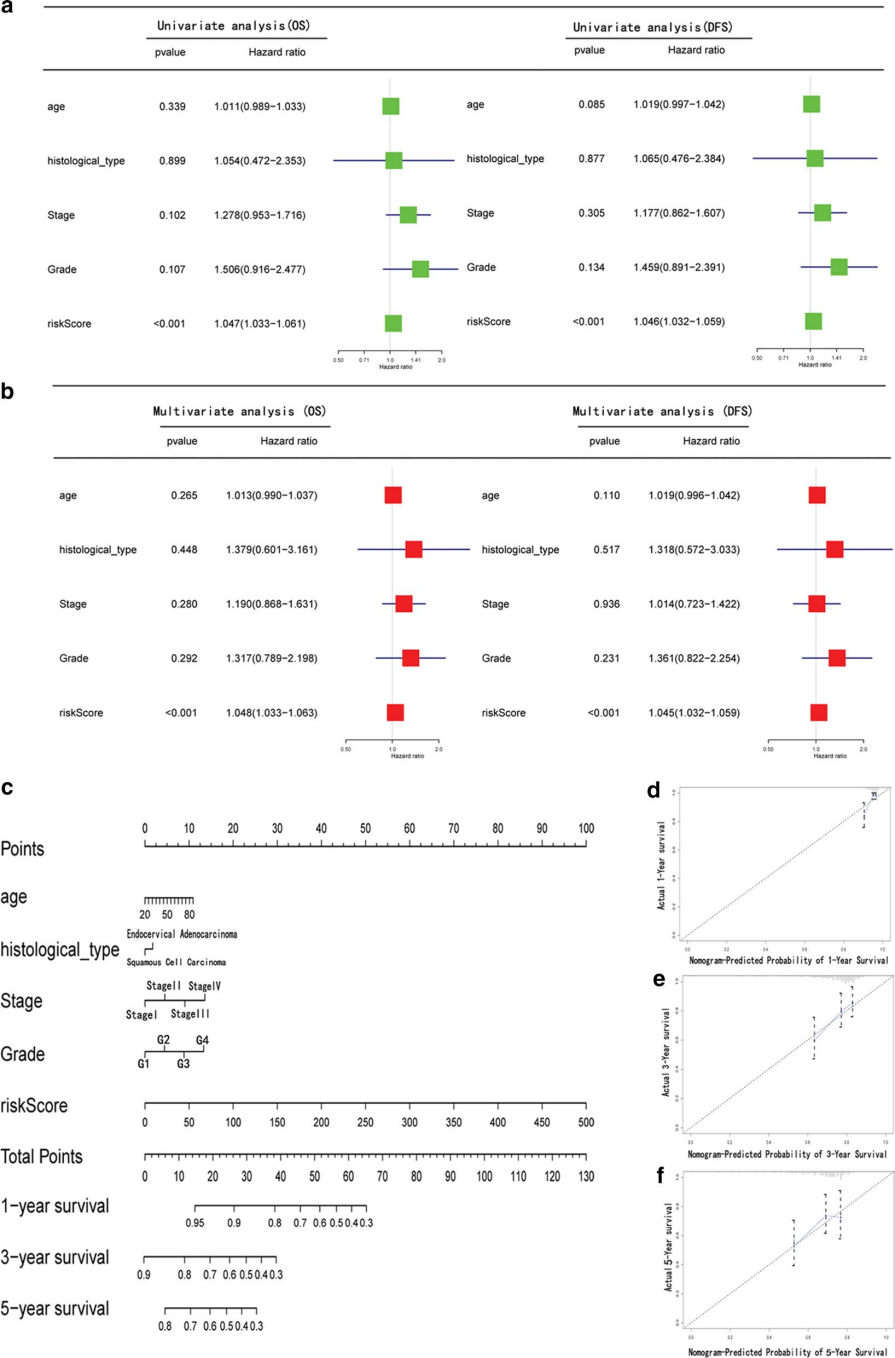
The prognostic value of risk scores and clinical characteristics in CESC. **a** Univariate analysis of risk scores and clinical characteristics that were simultaneously associated with OS and DFS. **b** Multivariate analysis of risk scores and clinical characteristics that were simultaneously associated with OS and DFS. **c** The nomogram for predicting probabilities of CESC patients with 1-, 3-, and 5- year OS. **d**–**f** The calibration plot for predicting patient 1-year, 3-year, and 5-year OS, respectively. Nomogram-predicted probability of survival is plotted on the *x*-axis; actual survival is plotted on the *y*-axis

Univariate and multivariate Cox regression methods were used and combined patient clinical characteristics (e.g., tumour stage and grade) to analyse whether the nine-AS event signature could be an independent predictor of survival in patients with CESC. We used the univariate Cox regression method to analyse the association of factors (e.g., risk score, stage, grade, age, and histological subtype) with OS and disease-free survival of the TCGA cohort. The results are shown in Fig. 4a.

After further multivariate Cox regression analysis, the risk score could still be used as a reliable and stable independent risk predictor in the CESC cohort (P < 0.01; Fig. 4b). Based on the multivariate analysis, we constructed a predictive nomogram (shown in Fig. 4c) that included risk scores and clinical characteristics (age, stage, grade, histological subtype). The OS model had a c-index value of 0.764. The calibration curve demonstrated that the predicted and observed values are satisfactorily consistent in predicting the 1-, 3-, and 5-year OS (Fig. 4d–f).

### Correlation network of splicing factors

Splicing factors can regulate AS events by binding to pre-mRNAs, affecting exon selection and choice of splicing site [31]. Splicing factors also have their own specific AS events. We identified 404 splicing factor genes through a literature search and Internet resources, and performed a survival analysis of these splicing factors to explore their involvement in the survival of patients with CESC. The results revealed that 39 splicing factors were obviously associated with OS in CESC patients. We obtained the expression levels of these splicing factors from previously downloaded RNA-Seq data of the CESC cohort. Next, in the CESC cohort, we analysed the correlation between the expression levels of these 39 splicing factors and the PSI values for each prognostic AS event (Spearman’s rank test, P < 0.05). An absolute value of correlation > 0.5 denoted significant correlation, indicating an interaction between the splicing factor and the splicing event. Thus, we identified 270 regulatory pairs of splicing factors and splicing events, including nine prognostic splicing factors and 130 survival-associated AS events (53 upregulated AS events and 77 downregulated AS events), to construct the correlation network.

**Fig. 5:**
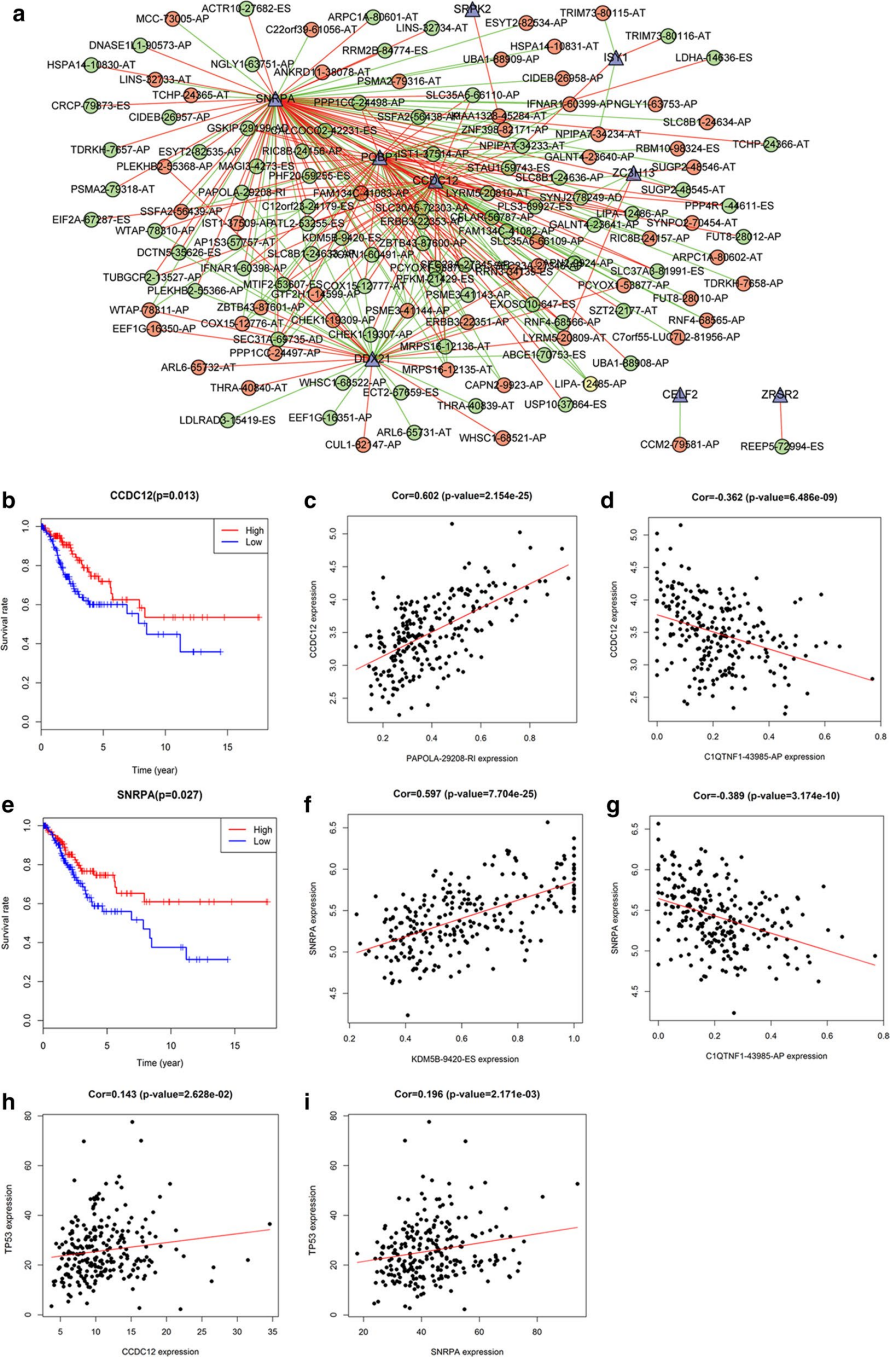
The splicing regulatory network of splicing factors and genes in the CESC cohort. **a** Regulatory network between the expression of prognostic splicing factors and the PSI values of prognostic AS events. The highlighted nodes in red indicate upregulation, and the highlighted nodes in green indicate downregulation. Splicing factors and AS events are indicated by triangles and ellipses, respectively. **b**, **e** Kaplan–Meier curves for splicing factors SNRPA and CCDC12 with high- and low-expression groups in CESC, respectively. **c**, **d** Correlations between the expression of splicing factor SNRPA and the PSI value of alternative splicing genes PAPOLA and C1QTNF, respectively. **f**, **g** Correlations between the expression of splicing factor CCDC12 and the PSI value of alternative splicing genes KDM5B and C1QTNF, respectively. **h**, **i** Correlations between the expression of both splicing factors CCDC12 and SNRPA and the expression of TP53, respectively

We subsequently used Cytoscape software to construct and visualize a regulatory network of splicing factors and AS events (Fig. 5a). In this regulatory network, both prognostic small nuclear ribonucleoprotein polypeptide A (SNRPA) and coiled-coil domain containing 12 (CCDC12) were identified as hub genes (Fig. 5b, e). We visualized the relationships between these two splicing factors and some prognostic AS events based on the R package “ggplot” (Fig. 5c, d, f, g). The analysis results showed that TP53 was associated with both splicing factors (Fig. 5h, i). A gene set enrichment analysis was conducted to identify the potential biological functions of CCDC12 and SNRPA related to the Kyoto Encyclopedia of Genes and Genomes pathways in CESC. We discovered that several cancer-related pathways (e.g., prostate cancer, small cell lung cancer, and pathways in cancer) were enriched (Additional file 3a, b).

### Molecular subtypes based on the nine AS events

**Fig. 6:**
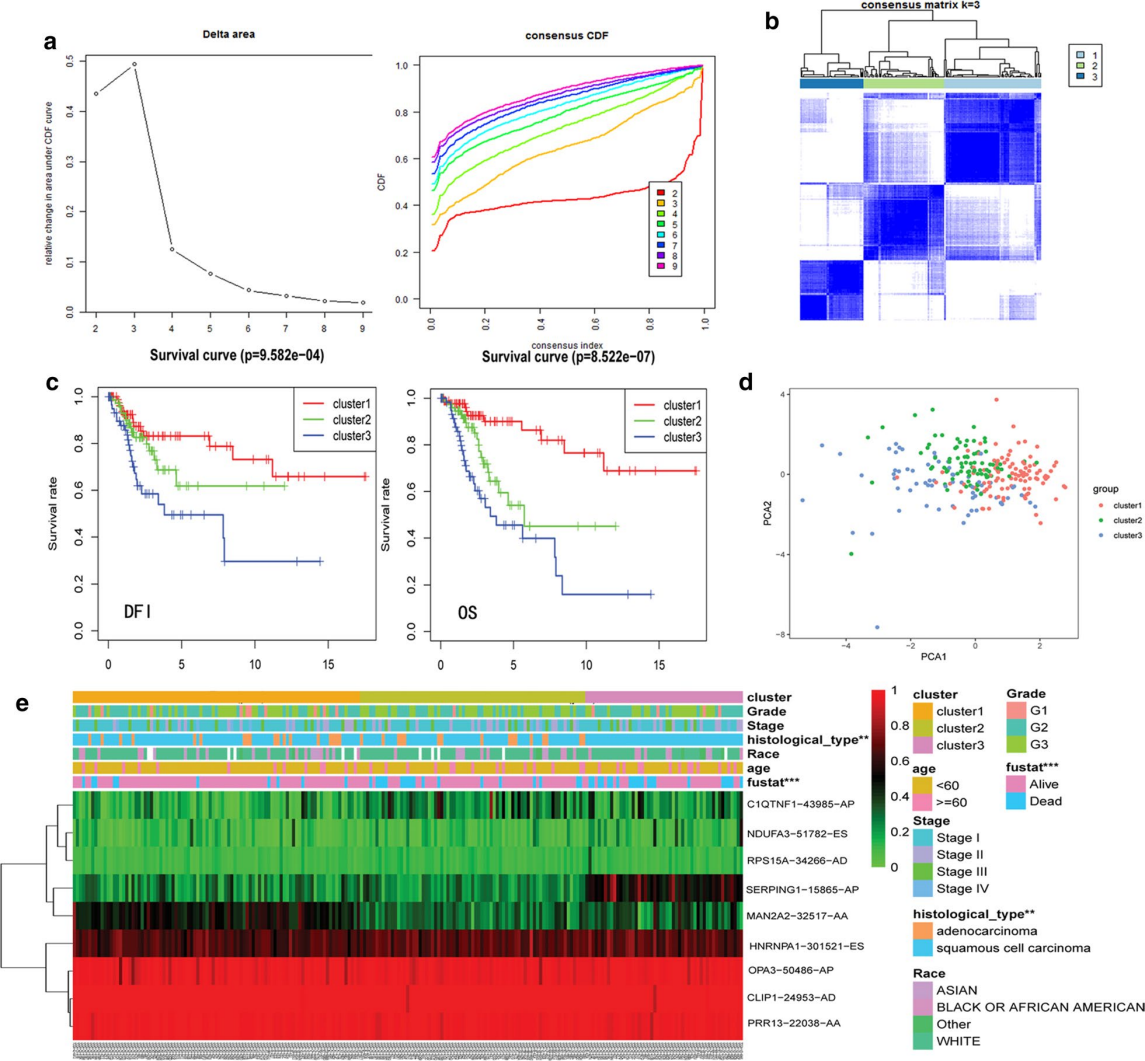
AS clusters associated with prognosis and molecular subtypes. **a** Consistent cumulative distribution function graph and the Delta Area Plot for different numbers of clusters (k = 2 to 9). **b** A consensus matrix heatmap defined three clusters. **c** Kaplan-Meier survival analysis for OS and DFS in different clusters of CESC patients. **d** Principal component analysis of different clusters. **e** Heatmap of the nine prognostic AS events sorted by cluster and the annotations associated with each cluster

We found that the nine AS events can reflect the prognosis of patients with cervical cancer. Hence, we performed a consensus unsupervised analysis of all samples based on these nine prognostic AS events. We determined the optimal number of clusters using the consistent cumulative distribution function graph and the Delta Area Plot (Fig. 6a). The final number of clusters was k = 3. Therefore, three clusters of patients were identified as follows: C1 (n = 98, 40.33%), C2 (n = 81, 33.33%), and C3 (n = 64, 26.34%). The consistency matrix heat map showed three clusters with significant interconnectivity (Fig. 6b). Subsequently, Kaplan–Meier analysis was used to evaluate the relationship between the clusters and prognosis.

The results indicated that the C3 cluster was associated with poor prognosis in overall survival (OS) and disease-free survival (DFS) (Fig. 6c). PCA was effectively able to distinguish the above molecular subtypes (Fig. 6d). The associations of clusters with the clinical data are shown in Fig. 6e. The distribution of clinical features in patients with CESC among the clusters was mostly random, except for histological types.

## Discussion

AS is a key factor in the cancer-related diversity of gene expression, and it is also the potential cause of non-genetic clonal expansion and epigenetic diversity [32]. This suggests that the therapeutic effect against tumours may rely on AS interactions. Studies have confirmed that AS plays a key role in the origin and progression of cervical cancer. Novikov et al. showed that the QKI-mediated histone variant MacroH2A1 AS regulated the proliferation of cervical cancer cells [33]. Studies have shown that cytokeratin 18 plays a role in the apoptosis of HeLa cells by regulating the transcription and AS of some genes in the apoptotic pathway [34]. Wu et al. analysed the AS of highly expressed genes in HeLa cells based on the single-cell RNA-seq platform. The results showed that at least two-thirds of the genes expressed more than two isoforms, indicating heterogeneity of AS in HeLa cells [35]. At present, studies investigating AS events in cervical cancer are based on single-gene research. Currently, there is no systematic study to combine this research with the clinical patient data for prognostic analysis. The implementation of SpliceSeq, termed an analysis pipeline, makes it possible to perform a systematic analysis of AS events using the RNA-Seq data in TCGA [36].

We identified variable splicing events with prognostic value using the AS data for CESC obtained from TCGA. Previous prognostic studies using TCGA data have shown that long non-coding RNAs (lncRNAs), microRNAs, and methylation data can serve as prognostic factors for cervical cancer. For example, Mao et al. predicted the survival of patients with CESC based on the risk scores constructed by 15 lncRNAs. The AUC of the ROC curve based on the optimal cut-off point was 0.946 [37]. Liang et al. used a model constructed by a ten-lncRNA signature to predict 5-year survival with an AUC value of 0.852 [38]. Shi et al. revealed an AUC value of 0.897 based on seven prognostic microRNAs [39]. Models constructed using methylation gene pair data have also shown favourable prognostic predictions [40, 41].

In our study, we analysed the predictive model constructed using a single AS model. The results showed that AA site events were more effective in identifying survival outcomes in patients with CESC than the other six types in the AS model. Our findings are consistent with those reported by other research studies [25]. In addition, we employed the risk scores established using the nine-AS event signature to assess the 5-year survival of patients with cervical cancer, with an AUC value of 0.87 in the ROC curve. An effective prognostic value was obtained by the use of this model. Moreover, we used the nine-AS event signature combined with some clinical pathological parameters to construct a predictive nomogram with an excellent predictive effect. To the best of our knowledge, most of the prognostic AS events we identified have not been reported, and this evidence is worthy of further experimental verification.

Moreover, we established a splicing-related network including the splicing factors and AS events. The splicing factors precisely regulate the splicing process by binding to the splicing regulatory sequences of specific genes [42]. Splicing factors are involved in the regulation of AS of pre-mRNAs associated with cancer progression and are thought to play a crucial role in cancer invasion and migration [43, 44]. This regulatory network of AS events can lead to a variety of abnormalities in genes associated with cancer progression. Song et al. revealed that CRKL regulated the AS of multiple genes that play a crucial role in tumorigenesis and the progression of cervical cancer [45].

The Ser/Arg-rich protein was originally discovered as a regulator of AS [42]. The Ser/Arg-rich protein family also plays a key role in regulating AS in cervical cancer. The cell-splicing factor serine and arginine-rich splicing factor 2 (SRSF2) regulated the expression of the human papillomavirus 16 oncoprotein [46]. In addition, SRSF3 controlled the alternative RNA splicing of human papillomavirus 18, thereby affecting gene expression [47]. Studies revealed that the splicing factor SRSF10 can mediate AS of IL1RAP to facilitate oncogenesis in cervical cancer [48].

In our constructed splicing factor regulatory network, we focussed on prognostic splicing factors and AS events. Both SNRPA and CCDC12 were identified as hub genes in the network. SNRPA is the protein component of the U1 small nuclear ribonucleic acid protein (U1 snRNP) complex, which inhibited spliced lncRNAs in the nucleus [49]. Dou et al. showed that SNRPA can enhance the growth of gastric cancer cells by modulating the expression of NGF [50]. Interestingly, the results of our study showed that the splicing factor SNRPA exerted a protective effect against cervical cancer. Contrary to its previously reported role in gastric cancer, we speculated that this is due to the regulation of AS events, which warrants further verification.

CCDC12 is located on chromosome 3 and is one of the proteins containing a coiled-coil region. CCDC12 is a prognostic gene in acute myeloid leukaemia [51]. Its mechanism in cervical cancer requires further investigation. In our study, CCDC12 and SNRPA were associated with TP53, which is the protein coding gene of oncoprotein P53, and is also associated with cancer-related pathways (e.g., prostate cancer and small cell lung cancer). In our splicing-related network, some different splicing events of the same gene play opposite roles in prognosis, and splicing factors can regulate AS events of different genes. Therefore, the regulatory effect of splicing factors depends on their own interaction with their cis-acting regulatory elements.

We divided patients with cervical cancer into three clusters based on nine AS events. Interestingly, the distribution of clinical features (e.g., different TNM staging, grade, and survival status) among the AS clusters was mostly random, except for histological types. Moreover, C3 was associated with poor prognosis in the OS and DFS analyses. In the consensus molecular subgroups, integrated genomic data were used to comprehensively cluster patients with cervical cancer. Three iClusters were identified, namely Keratin-low, Keratin-high, and Adenocarcinoma-rich [7]. Based on the 16 molecular features of the TCGA Pan-Cancer cohort study, 66% (114/173) of patients with cervical cancer were characterized with hyper-mutation and high immunity [10]. These findings indicated that the heterogeneity of cervical cancer is mostly determined according to its molecular characteristics. Our molecular classification of cervical cancer based on AS events can also effectively distinguish the histological types of cervical cancer and subsequently influence clinical prognosis.

To the best of our knowledge, this is the first and most comprehensive study to systematically identify and analyse survival-related splicing factors and regulated AS events in cervical cancer. Because of the stringent filter set used during the screening process, we are confident that the results related to the prognostic value of AS events are reliable. However, further research (molecular experiments and clinical trials) on these potential diagnostic biomarkers and therapeutic targets is required to validate these findings. In addition, the integrated analysis of prognostic splicing factors and survival-associated AS events provides a new perspective for studying the intrinsic mechanisms of splicing pathways involved in cervical cancer. A systematic study of prognostic AS events can assist physicians in better understanding the mechanisms of cervical cancer oncogenesis and progression, potentially providing a novel therapeutic strategy against cervical cancer.

## Conclusions

AS events affect the prognosis and biological progression of cervical cancer. The identified prognostic AS events and splicing regulatory networks can increase our understanding of the underlying mechanisms of cervical cancer and provide new therapeutic strategies.

## Supplementary information

**Additional file 1.** Overview of AS event profiling in CESC. (a) Schematic representation of seven types of AS events, including alternate acceptor (AA) site, alternate donor (AD) site, exon skip (ES), retained intron (RI), alternate promoter (AP), alternate terminator (AT), and mutually exclusive exons (ME). (b) The number of AS events and related genes in CESC. (c) The UpSet plot of interactions between the seven types of AS events in CESC. One gene may correspond to up to 6 types of alternative splicing.

**Additional file 2.** Forest plots and bubble chart for subgroup analyses of prognostic AS events in the CESC cohort. (a–f) Forest plots of hazard ratios (HRs) and bubble chart of P value for the top 20 prognostic AA, AD, AP, AT, ES, and RI events in CESC, respectively. (g) Forest plots of HRs and bubble chart of P values for prognostic ME events in CESC. (h) The UpSet intersection diagram shows seven types of prognostic AS events in CESC.

**Additional file 3.** Gene set enrichment analysis (GSEA). (a) GSEA enrichment plot showing the KEGG pathways associated with the CCDC12-high group. (b) GSEA enrichment plot showing the KEGG pathways associated with the SNRPA-high group (*p* value < 0.05).

## Data Availability

The data that support the findings of this study are openly available in the CESC cohort from the TCGA database (May 2019, https://portal.gdc.cancer.gov/) and the TCGA SpliceSeq database (http://projects.insilico.us.com/TCGASpliceSeq/).

## Abbreviations

AS: Alternative splicing
CESC: Cervical squamous cell carcinoma and endocervical adenocarcinoma
TCGA: The Cancer Genome Atlas
OS: Overall survival
PSI: Percent spliced in
AA: Alternate acceptor
AD: Alternate donor
AP: Alternate promoter
AT: Alternate terminator
RI: Retained intron
ES: Exon skip
ME: Mutually exclusive exons
LASSO: Least absolute shrinkage and selection operator
ROC: Receiver operating characteristic
AUC: Area under curve
lncRNAs: Long non-coding RNAs
