# Supplementary figures and images for: Comprehensive analysis of prognostic alternative splicing signature in cervical cancer
Source: Cancer Cell Int. 2020 Jun 8;20:221. doi: 10.1186/s12935-020-01299-4 (PMC7282181; doi:10.1186/s12935-020-01299-4)

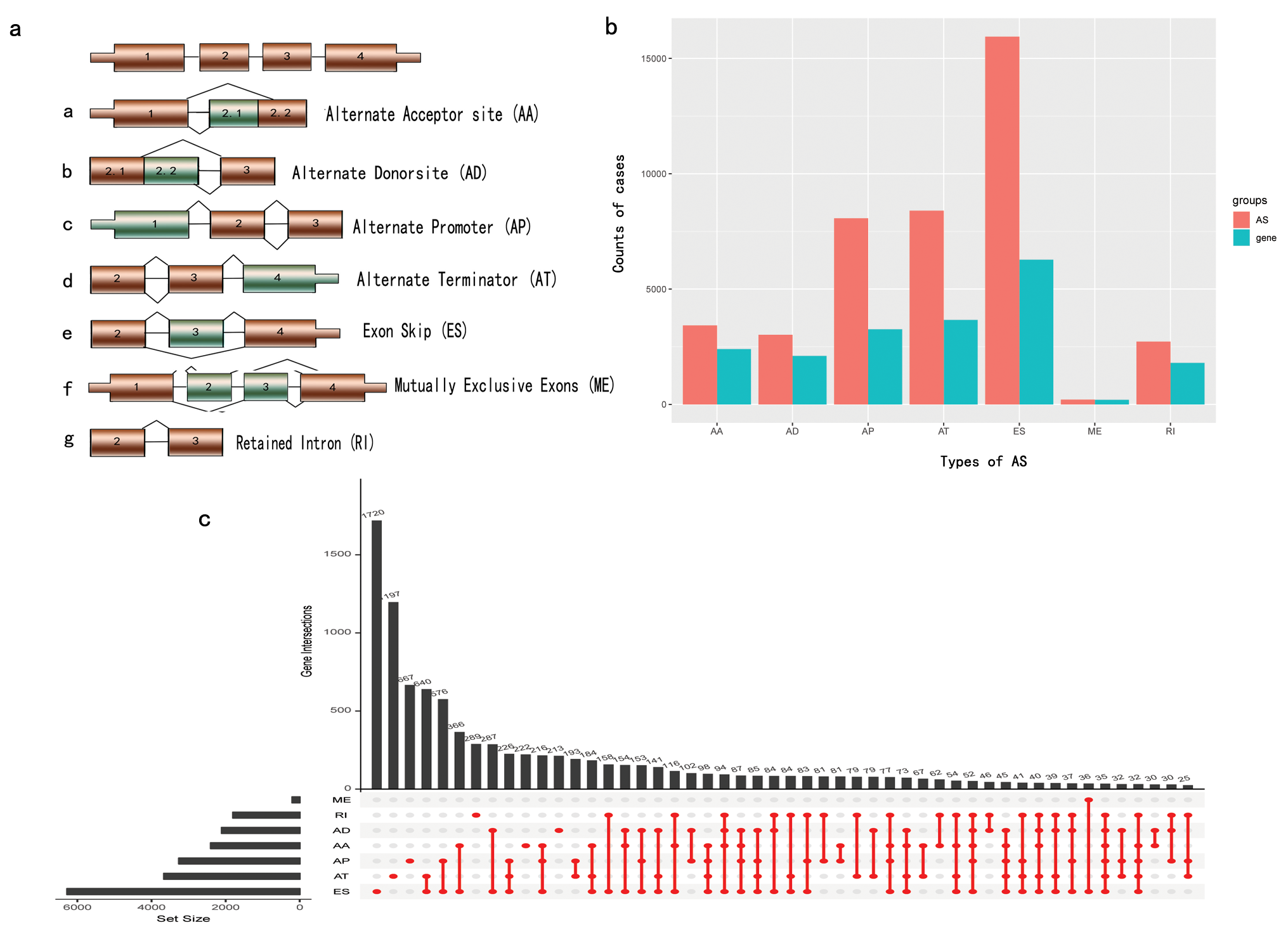

Supplement: Supplementary file 1 — Additional file 1. Overview of AS event profiling in CESC. (a) Schematic representation of seven types of AS events, including alternate acceptor (AA) site, alternate donor (AD) site, exon skip (ES), retained intron (RI), alternate promoter (AP), alternate terminator (AT), and mutually exclusive exons (ME). (b) The number of AS events and related genes in CESC. (c) The UpSet plot of interactions between the seven types of AS events in CESC. One gene may correspond to up to 6 types of alternative splicing. [file 12935_2020_1299_MOESM1_ESM.tif]

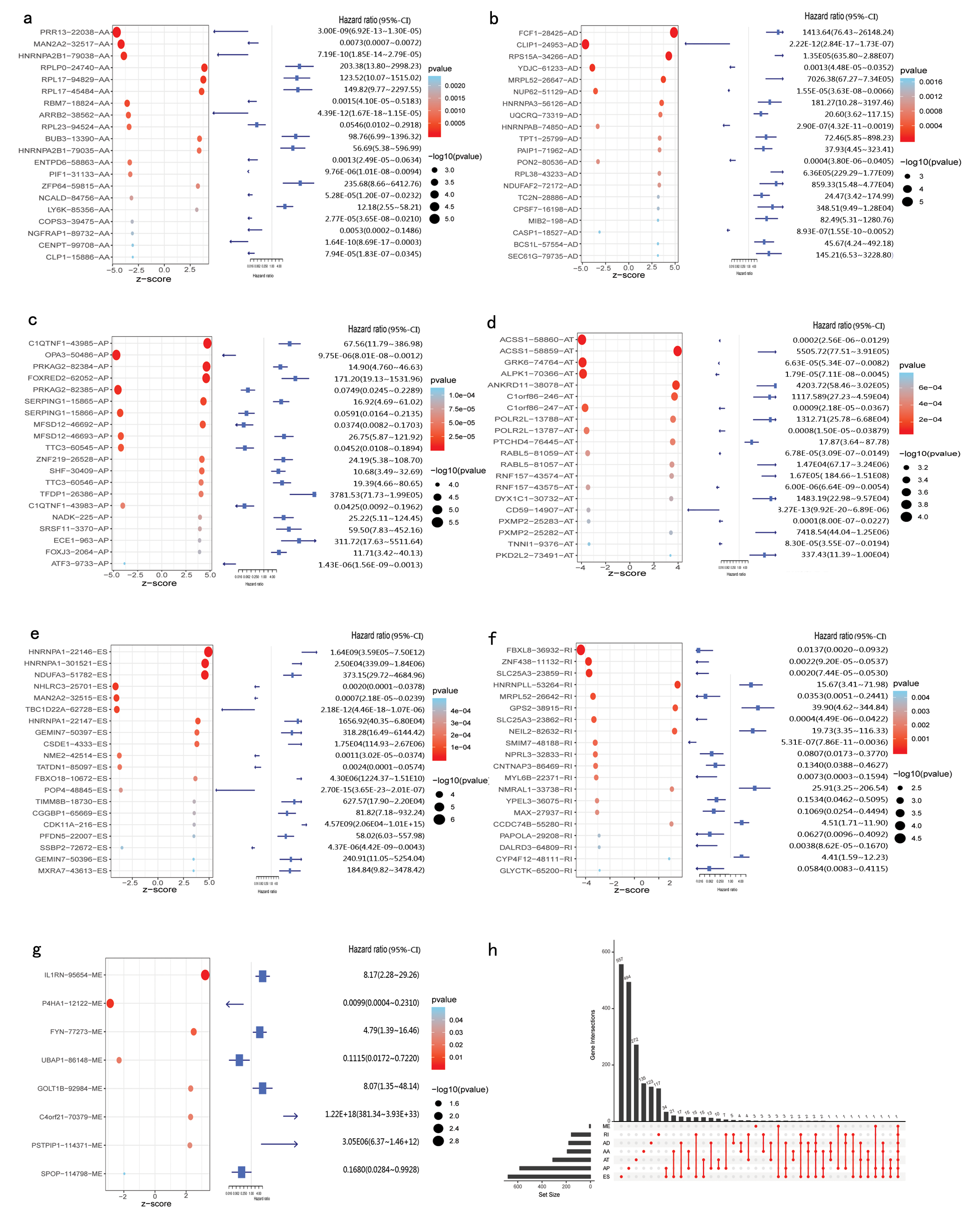

Supplement: Supplementary file 2 — Additional file 2. Forest plots and bubble chart for subgroup analyses of prognostic AS events in the CESC cohort. (a–f) Forest plots of hazard ratios (HRs) and bubble chart of P value for the top 20 prognostic AA, AD, AP, AT, ES, and RI events in CESC, respectively. (g) Forest plots of HRs and bubble chart of P values for prognostic ME events in CESC. (h) The UpSet intersection diagram shows seven types of prognostic AS events in CESC. [file 12935_2020_1299_MOESM2_ESM.tif]

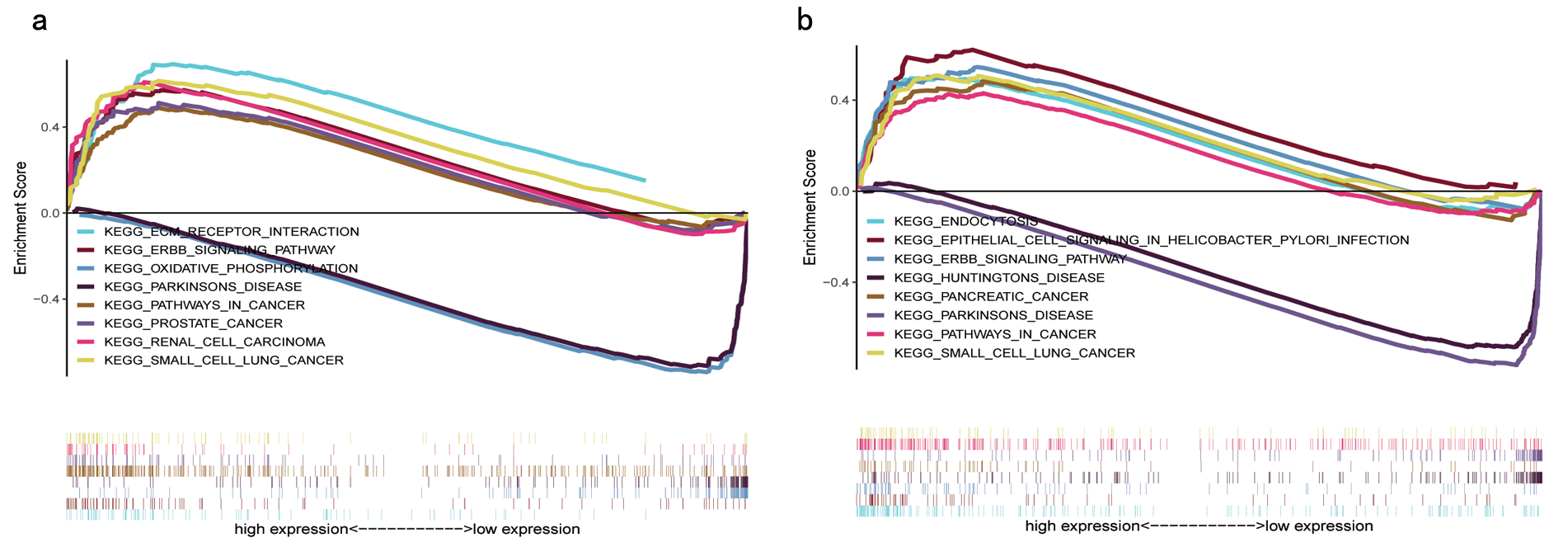

Supplement: Supplementary file 3 — Additional file 3. Gene set enrichment analysis (GSEA). (a) GSEA enrichment plot showing the KEGG pathways associated with the CCDC12-high group. (b) GSEA enrichment plot showing the KEGG pathways associated with the SNRPA-high group (p value < 0.05). [file 12935_2020_1299_MOESM3_ESM.tif]
